# Pediatric adrenocortical carcinoma

**DOI:** 10.3389/fendo.2022.961650

**Published:** 2022-10-31

**Authors:** Maran Ilanchezhian, Diana Grace Varghese, John W. Glod, Karlyne M. Reilly, Brigitte C. Widemann, Yves Pommier, Rosandra N. Kaplan, Jaydira Del Rivero

**Affiliations:** ^1^ Pediatric Oncology Branch, Center for Cancer Research, National Cancer Institute, Bethesda, MD, United States; ^2^ Developmental Therapeutics Branch, Rare Tumor Initiative, Center for Cancer Research, National Cancer Institute, Bethesda, MD, United States

**Keywords:** pediatric adrenal tumors, adrenocortical cancer (ACC), pediatric adrenocortical carcinoma, endocrine tumors, adrenal tumor

## Abstract

Adrenocortical carcinoma (ACC) is a rare endocrine malignancy of the adrenal gland with an unfavorable prognosis. It is rare in the pediatric population, with an incidence of 0.2-0.3 patients per million in patients under 20 years old. It is primarily associated with Li-Fraumeni and Beckwith-Wiedemann tumor predisposition syndromes in children. The incidence of pediatric ACC is 10-15fold higher in southern Brazil due to a higher prevalence of *TP53* mutation associated with Li-Fraumeni syndrome in that population. Current treatment protocols are derived from adult ACC and consist of surgery and/or chemotherapy with etoposide, doxorubicin, and cisplatin (EDP) with mitotane. Limited research has been reported on other treatment modalities for pediatric ACC, including mitotane, pembrolizumab, cabozantinib, and chimeric antigen receptor autologous cell (CAR-T) therapy.

## Introduction

Adrenocortical Carcinoma (ACC) is a rare endocrine malignancy with an overall unfavorable prognosis. It is rare amongst children, with an incidence of 0.2-0.3 patients per million, in patients under 20 years of age (1, 2), accounting for less than 0.2% of all pediatric malignancies (3). Pediatric ACC displays a bimodal distribution with peaks under the age of 5 and after 10 years and presents more frequently in females than males (4, 5). Pediatric ACC incidence is 10-15 times higher in southern Brazil, likely due to a higher prevalence of the R337H *TP53* mutation (6, 7). Pediatric ACCs are often linked to cancer predisposition syndromes, with most childhood ACCs associated with germline mutations (8, 9).

Pediatric patients with ACC often present differently than adult patients. One key difference from adult ACC is that pediatric ACCs are more often functional, often presenting with excess androgen production (5, 10, 11). Pediatric ACC patients have a 5-year survival rate reported to be between 30% to 70%, depending on disease presentation (11–13). Outcomes in patients with metastatic disease are poor, with a 5-year survival rate estimated to be less than 20% (1, 10, 11, 14, 15). Prognosis of childhood ACC is highly variable and difficult to predict in clinical practice (9).

## Genetics

ACCs that develop in children can either be sporadic or linked to a cancer predisposition syndrome. The genomic landscape of pediatric ACC is characterized by copy-neutral loss of heterozygosity of chromosomes 11 and 17, which is associated with germline *TP53* pathogenic variants, insulin-like growth factor-2 overexpression, and somatic mutations in *ATRX* and *CTNNB1* (16).

Pediatric ACCs are most commonly associated with Li-Fraumeni Syndrome, an autosomal dominant familial cancer syndrome associated with a number of malignancies including sarcoma, breast cancer, brain tumors, leukemia, lymphoma, and adrenocortical carcinomas (17–19). Li-Fraumeni Syndrome occurs in the context of germline mutations in the *TP53* tumor suppressor gene, which encodes for p53, a transcription factor that helps preserve genomic integrity and activates apoptosis in cells with DNA damage (20). Germline mutations in the gene encoding the p53 tumor suppressor located at 17p13.1 have been found in approximately 50% of children with ACC (21, 22). However, germline p53 mutations are much less common in adults with ACC (23).

In southern Brazil, there is a fifteen-fold increase in the incidence of pediatric ACC compared to other global populations (6). This is due to a unique germline missense mutation in *TP53* (R337H). This mutation exists at a high prevalence in this population (0.3%), but with a low penetrance of approximately 2%, which contrasts with the classical presentation of Li-Fraumeni Syndrome that presents with 100% penetrance (24–26). This R337H mutation affects the protein’s oligomerization domain, resulting in pH-dependent instability, predisposing these individuals to adrenocortical tumors (23, 26–29). Additionally, R337H mutation carriers are at increased risk of developing other tumors associated with Li-Fraumeni Syndrome, as well as adult ACC (12, 27). A newborn screening program for R337H mutations in southern Brazil exists and has been successful at early detection of ACCs in this population (24).

Pediatric ACCs have also been associated with Beckwith-Wiedemann syndrome, a systemic overgrowth disorder characterized by macroglossia, macrosomia, organomegaly, and abdominal wall defects (29, 30). This syndrome is a result of genetic defect caused by uniparental disomy in the 11p15 chromosomal region resulting in *IGF2* growth factor overexpression (31). Normally, the *IGF2* gene is only expressed from the paternal allele due to imprinting (32, 33), but disomy leads to expression from two copies of the gene. In instances of normal functionality, IGF2 activates type 1 IGF receptors, which stimulates cell survival (34). IGF2 plays a major role in fetal adrenal growth and steroidogenesis (35, 36). While IGF2 overexpression alone is not sufficient for adrenal carcinogenesis, it does promote ACCs (36, 37).

Other hereditary tumor syndromes associated with ACC that are less common in the pediatric population include multiple endocrine neoplasia 1 (MEN1), Lynch syndrome, familial adenomatous polyposis (FAP), Neurofibromatosis type 1 (NF1), and Carney complex (**Table 1**). MEN1 is an autosomal dominant hereditary tumor syndrome characterized by tumors affecting the parathyroid, pituitary, and pancreatic islet (38). Adrenal lesions occur in 20-55% of MEN1 patients, however, these lesions are usually adrenocortical adenomas or hyperplasia; ACC is extremely rare in this population (39). Lynch syndrome is an autosomal dominant inherited cancer predisposition syndrome characterized by an elevated risk of developing colorectal and endometrial cancers (40). The prevalence of Lynch syndrome amongst ACC patients is estimated to be 3.2% (41). FAP is an autosomal dominant hereditary tumor syndrome characterized by the development of adenomas in the rectum and colon followed by colorectal cancer if not treated at an early stage (42). Although rare, ACCs in patients with FAP have been reported in the literature (43–45). NF1 is an autosomal dominant genetic syndrome due to mutations in the *NF1* tumor suppressor gene and is characterized by café au lait spots, dermal and plexiform neurofibromas, pheochromocytomas, optic gliomas, and malignant peripheral nerve sheath tumors (46, 47). Rare instances of ACC in patients with NF1 have been reported in the literature (48–51). Carney complex is an autosomal dominant tumor syndrome characterized by skin pigmentary abnormalities, myxomas, endocrine tumors, and schwannomas (52). ACC in the setting of Carney complex is very rare, but has been reported in two cases in the literature (53–55).

**Table 1:** Hereditary tumor syndromes associated with ACC.

Syndrome	Associated Gene mutations	Major clinical features	Prevalence %-ACC
Li-Fraumeni syndrome	*TP53*	Breast cancer, leukemia, lymphoma, brain tumors, sarcomas, adrenocortical carcinoma	3-7% in adults 50-80% in children
Beckwith-Wiedemann syndrome	*IGF2, CDK1C*, *H19* locus changes on 11p15	Wilms tumor, hepatoblastoma and neuroblastoma	<1%
Multiple Endocrine Neoplasia 1	*MEN1*	Hyperparathyroidism, pituitary tumors, parathyroid tumors, pancreatic neuroendocrine tumors (PNETs), collagenoma, angiofibroma	1.4%
Lynch syndrome	*MSH2, MSH6, MLH1, PMS2*	Colorectal carcinoma, endometrial carcinoma, ovarian cancer, pancreatic cancer, brain cancer	3%
Familial adenomatous polyposis	*APC*	Colorectal cancer, duodenal carcinoma. Thyroid cancer, desmoid tumor	<1%
Neurofibromatosis type 1	*NF1*	Malignant peripheral nerve sheet tumor, pheochromocytoma, neurofibroma, optic glioma	<1%
Carney complex	*PRKAR1A*	Primary pigmented nodular adrenocortical disease (PPNAD), Sertoli cell tumors, thyroid adenoma, myxoma, pituitary tumors, schwanoma	<1%

## Clinical characteristics

Pediatric ACC displays a bimodal age distribution with peaks under the age of 5 and after 10 (4), with almost half (46%) of diagnoses occurring in children less than 4 years of age (1). There is a female preponderance for this condition both in childhood and in adulthood. Previous studies have shown the growth-promoting role of estrogen on the ACC cell line NCI-H295 and may explain the basis for this predisposition (56). There is a 2:1 female to male ratio amongst diagnoses of pediatric ACC (1).

Based on international TNM staging, approximately 44% of patients are stage I, 25% stage II, 13% stage III, and 17% stage IV (57). However, differences may exist based on age. Based on data from a SEER study of pediatric ACC patients, 52% of all staged patients presented with localized tumors; however, this was 76% in those aged 4 years and younger compared to 31% in those over the age of 4 (1). Additionally, the same study showed a significantly greater tumor size in older patients. Metastases are found in approximately 25% of pediatric ACC patients (57). The majority of metastases are found in the lungs and liver (14, 57). Other reported sites of metastases include the bone, kidneys, and CNS (57).

Pediatric ACCs are almost always functional, with approximately 95% of tumors exhibiting hormone production, compared to less than 50% of adult ACCs (11, 58, 59). Consequently, diagnoses in children usually follow the presentation of symptoms (12). Amongst all pediatric adrenocortical tumors, the most common presentation is virilization due to excess androgen secretion alone or in combination with hypercortisolism in over 80% of patients (11, 12). Other presentations include Cushing syndrome (15-40%), feminization or gynecomastia due to excess estrogen production (7%), hyperaldosteronism (1-4%), or a mixture of symptoms (60). Cushing’s syndrome without virilization is uncommon (5.5%) (11). Approximately 10% of pediatric patients with ACC have nonfunctional tumors; they are uncommon amongst young children, and are usually only found in adolescents (11, 60).

Michalkiewicz et al. created a registry for pediatric ACCs and provide a descriptive analysis of 254 patients registered on the International Pediatric Adrenocortical Tumor Registry. 254 patients younger than 20 years of age with newly diagnosed or previously treated ACCs were registered. The most common presenting sign (84.2%) was virilization. Cushing’s syndrome without virilization was uncommon (5.5%). Tumors were completely resected in 83% of patients. Patients with disseminated or residual disease received mitotane, cisplatin, etoposide, and/or doxorubicin, and rarely, radiation therapy. At a median follow-up of 2 years and 5 months, 157 patients (61.8%) survived without evidence of disease and 97 patients (38.2%) had died. The 5-year event-free survival estimate was 54.2%. It was concluded that childhood ACCs occur predominantly in females and almost always causes clinical signs. Complete resection is required for cure. Residual or metastatic disease carries a poor prognosis (11). Wieneke’s index has been validated in some studies is a histopathological tool which have shown to predict clinical outcome in pediatric ACC patients (**Table 2**) (61). Pediatric patients with age <4 y on presentation shows favorable prognosis while metastasis at presentation have guarded prognosis (14).

**Table 2A:** Wieneke’s criteria.

Wieneke’s Criteria for malignancy	
Tumor weight >400g
Tumor size >10.5cm
Extension into periadrenal soft tissues and/or adjacent organs
Invasion into vena cava
Venous invasion
Capsular invasion
Presence of tumor necrosis
>15 mitoses per 20 high power field (400X)
Presence of atypical mitotic figures

**Table 2B:** Clinical outcomes based on Wieneke’s criteria.

**Two criteria**	Benign long-term clinical outcome
**Three criteria**	Intermediate/atypical/uncertain malignant potential
**Four or more criteria**	Poor clinical outcome

## Treatment

The treatment for pediatric ACC has largely been extrapolated from adult ACC. The European Cooperative Study Group for Pediatric Rare Tumors has published consensus guidelines for the diagnosis and treatment of childhood adrenocortical tumors, derived from the guidelines and data obtained from adult studies (11). Surgery is the primary form of therapy, with an aggressive surgical approach recommended if feasible (62). Results from a recent prospective single-arm risk stratified interventional study with 77 patients showed that surgery has been the mainstay treatment and had shown to have excellent prognosis in stage I ACC (86.2% 5-year event free survival) (63). However, tumor spillage is a frequent concern in these patients with its occurrence in approximately 21% of initial resections and 43% of resections after recurrence (11). Due to this risk of tumor rupture, laparotomy and a curative procedure are recommended, rather than a fine needle aspiration (64). An open adrenalectomy is the standard care for resection of ACC, as laparoscopic resections are associated with a high risk of rupture and peritoneal carcinomatosis (62, 65).

Mitotane is a commonly used single agent in the adjuvant setting following a complete resection of ACC in the adult population and is approved for the treatment of ACC (66). Mitotane inhibits steroidogenesis and has direct adrenolytic functions, inducing permanent atrophy of the fasciculata and reticular zones of the adrenal cortex (67, 68). Mitotane has shown to improve outcomes in the intermediate risk pediatric ACC population if it can be given for more than 6 months and if therapeutic levels (greater than 14mg/L) can be attained (14, 69). In the pediatric ACC population, it has been shown to improve outcomes in stage III and IV, although it is poorly tolerated (63). In a review of 11 children with advanced ACC who were treated with mitotane and a cisplatin based chemotherapeutic regimen, 7 patients showed measurable responses, suggesting that neoadjuvant use can be considered in patients where complete surgical resection is not an option (68). Additionally, a retrospective analysis of 177 patients with adrenocortical carcinomas showed a significant increase in recurrence-free survival amongst patients who received adjuvant mitotane therapy (66). In a interventional open label Phase III trial with pediatric ACC, Stage III ACC patients was shown to have a good clinical outcome with adjuvant chemotherapy with EDP and mitotane but was associated with toxicity. An optimal treatment regimen need to be curated for these patients (63).

Pembrolizumab is a molecular therapy that targets the programed death receptor 1(PD-1) on lymphocytes and has shown to be effective in tumors expressing PD-L1. In a study of Pembrolizumab therapy in pediatric patients with PD-L1 positive tumors, two out of four pediatric ACC patients enrolled showed a partial response (70).

Cabozantinib is a multi-tyrosine kinase inhibitor that has been studied in ACC patients. In a study of 16 patients with advanced ACC treated with cabozantinib, three patients developed a partial response and five patients had stable disease with a median progression free survival of 16 weeks and a median overall survival of 58 weeks (71). Currently, there is an ongoing trial for Cabozantinib-S-Malate for patients with rare tumors including pediatric ACC (**Table 3**) (72).

**Table 3:** Clinical trials for pediatric ACC.

Therapy	Study Design	# of Patients	Eligibility Criteria	NCT	Status
Cisplatin	Phase 3	78	Histologically confirmed ACC	NCT00304070	Active, Not recruiting. Results available
Cabozantinib-S-Malate	Phase 2	109	Ewing sarcoma, rhabdomyosarcoma, osteosarcoma, Wilms tumor, medullary thyroid carcinoma, renal cell carcinoma, hepatocellular carcinoma, hepatoblastoma, adrenal cortex carcinoma, pediatric solid tumors with specified molecular alterations	NCT02867592	Active, Not recruiting, Results pending.
B7-H3-CAR T Cells	Phase 1	32	Solid tumor, ≤21 years old	NCT04897321	Recruiting

B7-H3-specific chimeric antigen receptor autologous T-Cells (CAR-T) have shown success in treating patients with relapsed pediatric acute lymphoblastic leukemia as well as *in vivo* success in solid tumor types (73). Currently there is a B7-H3-CAR-T cell trial testing the viability of this therapy in pediatric solid tumor types, including pediatric ACC (**Table 3**).

The use of radiation therapy in pediatric ACC patients has not been widely studied. Since most pediatric ACC cases carry TP53 mutations that predispose them to cancer, radiation can increase the likelihood of developing a secondary tumor. Driver et al., reported that amongst five long term survivors of pediatric ACC in their study, three died of secondary sarcomas that arose in the radiation field (74).

## Future direction/therapeutics

**Scheme 1:**
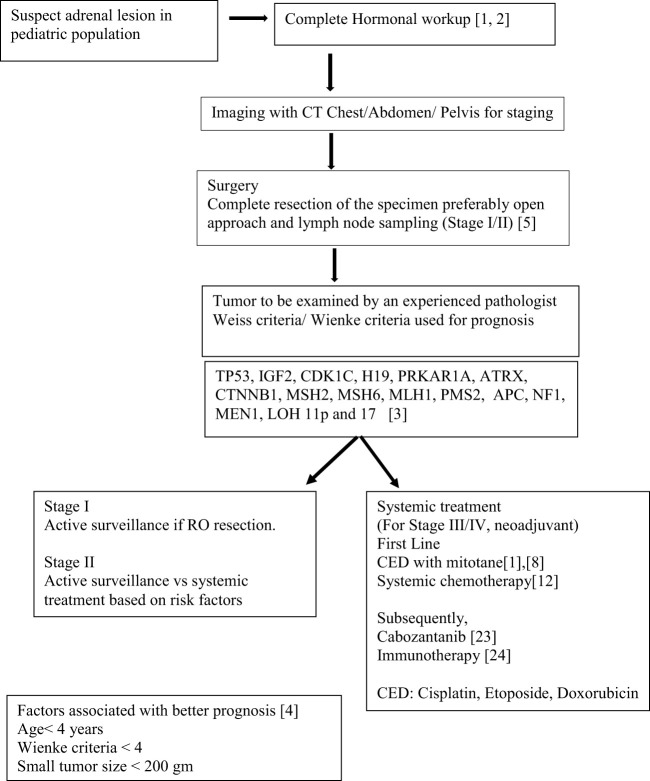
Schema showing Current guidelines and therapeutics strategies.

Understanding the oncogenesis and tumor biology of ACC in pediatric patients has been fallen behind compared to adult ACC. The treatment protocols are primarily based on the data from adult patients with adrenocortical cancer. Surgery with lymph node dissection, chemotherapy with EDP (mitotane, cisplatin, etoposide, and/or doxorubicin) and radiation in some cases are the current preferred treatment options in pediatric ACC (**Scheme 1**).

It has been shown that the similar histopathology in pediatric and adult populations have different prognosis and the prognostic markers used in adult ACC might not be applicable for pediatric cases (75). Pediatric ACC arises from the fetal zone of adrenal gland and almost always presents with signs of hormone excess (14). As described previously germline mutation (p.R337H) of the *TP53* gene and *IGF1R* overexpression is seen specifically in pediatric population (75). The expression of *FATE1* gene has a negative prognostic significance in adult but not in pediatric population. Pediatric ACC is also more frequently associated with cancer predisposition syndromes (76). Ki-67 index and Weiss score are not predictive in pediatric ACC (7). Moreover, even with the differences with adult ACC, the current ongoing trials for adrenocortical cancer may show some future directions for treatment of pediatric ACC. Unfortunately, however, much of the preclinical and clinical trial data we have comes from adult ACC and no pediatric cell line for this tumor exist, limiting our ability to study the pediatric ACC population (77). As such, much of the investigation into treatment modalities for pediatric ACC patients has been extrapolated from adult in-vitro and in-vivo studies.

Targeted molecular therapies have proven to be efficacious in a number of malignancies. Their use in ACC has been studied with limited success. IGF2 overexpression has been found in approximately 90% of ACC cases, making it a potential target for precision molecular therapy (78). Additionally, in pediatric ACC, IGF1R overexpression is associated with a worse prognosis (75, 79). Preclinical studies of IGF2/IGF1R antagonists in ACC xenograft models have been promising, showing a dose dependent growth inhibition (80). Unfortunately, targeted therapies for these targets have not yet been substantiated *in vivo*. The IGF1R inhibitor lisitinib failed to show improvement in overall or progression free survival in an adult Phase III clinical. However, 5/90 patients showed a stabilization of disease (81).A phase II trial on the IGF1R inhibitor cixutumumab in combination with mitotane was terminated due to slow accrual and limited efficacy (82).

The activation of β-catenin signaling has been found in approximately 40% of ACC tumors (83, 84). Studies done on the NCI-H295R in-vitro model of ACC have shown that inhibition of β-catenin can inhibit tumor proliferation and promote apoptosis (76, 85, 86). However, the clinical utility of Wnt/β-catenin signaling inhibitors has not yet been demonstrated. Wnt/β-catenin signaling is essential for stem/progenitor cell maintenance (83). As such, Wnt/β-catenin signaling inhibition can result in a number of on-target toxicities, which has prevented these agents from advancing to phase III clinical trials (87, 88).

The Ki-67 proliferation index of has demonstrated a major prognostic role in ACC (89). This finding combined with genomic findings in ACC tumors suggest a role for cell cycle activation in advanced ACC (78, 90, 91) The cell cycle inhibitors, gemcitabine, capecitabine, and 5-fluorouracil have shown moderate efficacy and tolerability in patients with advanced ACC. Further investigation of broad and targeted kinase cell cycle inhibitors may be warranted in patients with advanced ACC.

Promising data exists supporting the use of certain modalities of radiotherapy for treating ACC. A study on Iodine-131 Iodometomidate (^131^I MTO) targeted radionuclide therapy demonstrated one partial response and five patients with stable disease in a cohort of 11 patients with unresectable ACC (92). In ACC patients with somatostatin expressing tumors, Yttrium-90/^177^Lu-DOTATOC has shown potential as a treatment option. In a study of 19 patients with advanced ACC, eight patients showed radiometabolic uptake of Yttrium-90/^177^Lu-DOTATOC and two patients showed strong uptake in addition to overall disease control lasting 4 and 12 months (93).

The use of single cell sequencing of tumor specimen from primary and metastasis in larger cohort of patient population to understand of heterogeneity in ACC and use of liquid biopsies will help in more personalized treatment approach for pediatric ACC. The continuation of global consortiums and international collaborations like International Pediatric Adrenocortical Tumor Registry (IPACTR) are necessary to build on the existing knowledge of pediatric ACC (11).

## Conclusions

ACCs are a rare tumor in the pediatric population with a poor prognosis. Current treatment algorithms for pediatric ACC patients are based on research in adult populations. Investigation of cisplatin, cabozantinib and CAR-T cell therapy for pediatric ACC patients is ongoing. Research of treatment modalities for childhood ACC is limited and the need for more investigation into the treatment of this unique population exists. Due to the differences in ACC between pediatric and adult populations, we believe additional research in the treatment of childhood ACC is warranted.

## Author contributions

MI and DV: prepared the manuscript. JG, KR, RK, BW, YP, JR: reviewed the article and suggested edits. All authors contributed to the article and approved the submitted version.

## Funding

This project has been funded in whole or in part with federal funds from the National Cancer Institute, National Institutes of Health, under contract number HHSN261200800001E and the MyPART: My Pediatric and Adult Rare Tumor Network - Cures ZIA BC 011852. The content of this publication does not necessarily reflect the views or policies of the Department of Health and Human Services, nor does mention of trade names, commercial products, or organizations imply endorsement by the US Government. Funded by the Division of Intramural Research National Cancer Institute.

## Conflict of interest

The authors declare that the research was conducted in the absence of any commercial or financial relationships that could be construed as a potential conflict of interest.

## Publisher’s note

All claims expressed in this article are solely those of the authors and do not necessarily represent those of their affiliated organizations, or those of the publisher, the editors and the reviewers. Any product that may be evaluated in this article, or claim that may be made by its manufacturer, is not guaranteed or endorsed by the publisher.
